# Non-invasive monitoring of changes in rabbit hearts with aging using MR microscocpy

**DOI:** 10.1186/1532-429X-13-S1-P361

**Published:** 2011-02-02

**Authors:** Min-Sig Hwang, Katja E Odening, Bum-Rak Choi, Gideon Koren, Stephen J Blackband, John R Forder

**Affiliations:** 1 McKnight Brain Institute, University of Florida, Gainesville, FL, USA; 2 Cardiovascular Research Center, The Rhode Island Hospital, Alpert Medical School of Brown University, Providence, RI, USA

## Background

Loss of myocytes followed by compensatory hypertrophy of the remaining cells and interstitial fibrosis are common hallmarks observed in aging hearts [1,2]. The cardiac conduction system is also subject to alterations with aging, resulting in reduced thresholds to diseases [3]. Biopsy is an accurate and informative means to delineate the age-related changes. However, it is destructive and requires intensive labor, often with severe complications for 3D reconstruction. The aim of this study was to investigate non-invasively morphological changes in aging heart using MR microscopy.

## Materials and method

### Isolated heart preparation

Hearts (n = 5) of New Zealand White male rabbits (2-5 kg) were isolated and fixed *in situ* according to the approved animal protocol. Young hearts (n = 2) were 6 month ~ 1 year old. Old hearts were 3 ~ 4 years old.

### MRI

MR experiments were performed on a 17.6 T / 89 mm vertical wide-bore magnet (Bruker Instruments, Billerica, MA). Three dimensional MR microscopy data were collected using a fast gradient pulse sequence, achieving a voxel resolution of 35 x 35 x 82 μm^3^. High angular resolution diffusion microscopy (HARDM) using 21 directions was performed with a standard PGSE sequence, achieving an in-plane resolution of 60 μm^2^ with a slice thickness of 600 μm. The b-value was 1000 s/mm^2^_._

### Data analysis

Volume rendering of the 3D MR data sets was performed using ImageJ (ver. 1.31, http://rsbweb.nih.gov/ij/). The tensor processing of HARDM data sets was conducted using fanDTasia™ (©2008, http://www.cise.ufl.edu/~abarmpou/ ) and MATLAB (Mathsoft, Cambridge, MA).

## Results

**Figure 1:**
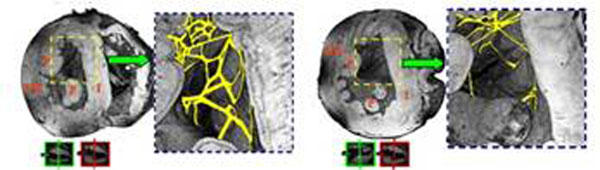
Volume rendered transverse images and manual separation (magnified) of the free-running Purkinje fiber network in the LV of a young rabbit heart (left) and an old rabbit heart (right). Lines and arrows in the green and red boxes indicate where sectioning occurred and viewer was located. Interseptal thickness is approximately 3.93 mm (young) and 4.82 mm (old). I: ventricular interseptum, P: papillary muscle, FW: free wall.

**Figure 2:**
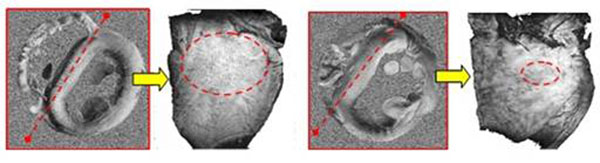
Tensor component (D_vs_) map from HARDI and a volume rendered image of the interseptum of a young rabbit heart (left) and an old rabbit heart (right). Dotted lines (red) indicate where sectioning occurred and dotted circles confine regions where the stripe pattern in observed.

Aging in the rabbit heart results in significantly increased interseptal thickness (Fig.1). Volume rendered transverse images of the apical half suggest that the free-running Purkinje fiber network in the LV cavity may significantly alter with aging (Fig. 1). Dense reticular net observed in young ventricles becomes sparse with aging and the free running Purkinje fibers become thinner. From HARDM, aging in rabbit heart appears to be associated with increased radial diffusivity, which may arise from cellular hypertrophy (data not shown). A tensor component map of a young heart (D_yz_) shows a well defined stripe pattern extending transmurally in the interseptum and left ventricular freewall (Fig. 2, left). In contrast, this highly directional structure was decreased in aged hearts (Fig.2, right). A volume rendered image of interseptum shows that the stripe pattern has a longitudinal direction and appears to exist in the basal half of the LV.
